# Burden and consequence of birth defects in Nepal-evidence from prospective cohort study

**DOI:** 10.1186/s12887-021-02525-2

**Published:** 2021-02-15

**Authors:** Prajwal Paudel, Avinash K. Sunny, Rejina Gurung, Abhishek Gurung, Honey Malla, Netra B. Rana, Nawaraj KC, Ram Narayan Chaudhary, Ashish KC

**Affiliations:** 1 Paropakar Maternity and Women’s Hospital, Ministry of Health and Population, Kathmandu, Nepal; 2 Research Division, Golden Community, Lalitpur, Nepal; 3 Lumbini Hospital, Provincial Ministry of Health and Population, Butwal, Nepal; 4 Surkhet Provincial Hospital, Provincial Ministry of Health and Population, Surkhet, Nepal; 5 Koshi Provincial Hospital, Provincial Ministry of Health and Population, Morang, Nepal; 6 Department of Women’s and Children’s Health, Uppsala University, Dag Hammarskjölds väg 14B, Uppsala, Sweden

**Keywords:** Birth defects, Nepal, Prevalence, Risk factor, Mortality

## Abstract

**Background:**

Every year an estimated 7.9 million babies are born with birth defect. Of these babies, more than 3 million die and 3.2 million have disability. Improving nationwide information on prevalence of birth defect, risk factor and consequence is required for better resource allocation for prevention, management and rehabilitation. In this study, we assess the prevalence of birth defect, associated risk factors and consequences in Nepal.

**Method:**

This is a prospective cohort study conducted in 12 hospitals of Nepal for 18 months. All the women who delivered in the hospitals during the study period was enrolled. Independent researchers collected data on the social and demographic information using semi-structured questionnaire at the time of discharge and clinical events and birth outcome information from the clinical case note. Data were analyzed on the prevalence and type of birth defect. Logistic regression was done to assess the risk factor and consequences for birth defect.

**Results:**

Among the total 87,242 livebirths, the prevalence of birth defects was found to be 5.8 per 1000 live births. The commonly occurring birth defects were anencephaly (3.95%), cleft lip (2.77%), cleft lip and palate (6.13%), clubfeet (3.95%), eye abnormalities (3.95%) and meningomyelocele (3.36%). The odds of birth defect was higher among mothers with age < 20 years (adjusted Odds ratio (aOR) 1.64; 95% CI, 1.18–2.28) and disadvantaged ethnicity (aOR 1.78; 95% CI, 1.46–2.18). The odds of birth asphyxia was twice fold higher among babies with birth defect (aOR 1.88; 95% CI, 1.41–2.51) in reference with babies without birth defect. The odds of neonatal infection was twice fold higher among babies with birth defect (aOR 1.82; 95% CI, 1.12–2.96) in reference with babies without birth defect. Babies with birth defect had three-fold risk of pre-discharge mortality (aOR 3.00; 95% CI, 1.93–4.69).

**Conclusion:**

Maternal age younger than 20 years and advantaged ethnicity were risk factors of birth defects. Babies with birth defect have high risk for birth asphyxia, neonatal infection and pre-discharge mortality at birth. Further evaluation on the care provided to babies who have birth defect is warranted.

**Funding:**

Swedish Research Council (VR).

## Introduction

Birth defects are anomalies in morphogenesis during early foetal life resulting in structural, behavioural, functional and metabolic disorders that can be detected prenatally, at birth or later in infancy [1]. Globally, more than 3 million babies with birth defect die within the first 28 days of life and a majority of the survivors suffer with disabilities [2, 3]. Lower- and middle-income countries (LMIC) carry the major burden (>90%) of these anomalies which are least prioritized as compared to other causes of neonatal and infant mortality for prevention and management [2, 3]. There is a high risk of mortality due to congenital heart disease and neural tube defects globally [2, 4].

South-East Asia has higher prevalence of cleft lip and palate [5], whereas neural tube defects is highly prevalent in Mexico, Central America, and India [3]. In Nepal in 2010, among the estimated 600,000 born 40,000 births were are born with malformations with cleft lip and palate, neural tube defects, congenital heart disease as the commonest conditions [5, 6]. The prevalence of birth defect is widely variable ranging from 0.3 to 7% implicating various known and unknown factors interplaying differently in varied time and geographical location [7]. Often, these factors are recognised as genetic in origin (10–30%), environmental (5–10%), multi-factorial (20–35%) and unknown (30–45%) [8]. Primarily these factors may affect the developing foetal organs during the first trimester while undergoing crucial stages of formation passing through stages of fertilization, implantation and organ formation [1, 8]. Identification of several maternal factors for birth defects like maternal age, lack of nutritious diet, no use of peri-conceptional folic acid, alcohol and tobacco consumption, exposure to pesticides and X-rays, and infection signifies the role of effective antenatal care (ANC) counselling and screening [9, 10].

To the best of the present evidence, therapy, medication, surgery, or assistive technology are different services available for management of birth defects and in most cases essential paediatric surgery can avert early mortality and long-term disability [11]. The Human capital approach to repair cleft lip and Palate in LMICs and favourable outcomes in High income Countries (HICs) in Gastrointestinal and other anomalies have refuted the traditional perception about Paediatric surgery [12]. Prenatal diagnostic techniques, genetic counselling, and access to termination of Pregnancy remain the cornerstone to curtail neonatal mortality and still birth [13].

In LMICs, lack of diagnostic and national screening programmes has led to paucity of nationally representative data, a major hurdle for in-depth understanding of the epidemiology [14]. In Nepal, no protocol regarding management and timely service provision to babies born with birth defects exists except for sporadic programmes for cleft lip and palate repair or few organisations providing care to disabled children. To further accelerate the reduction of 2016 neonatal mortality rate of 21 per 1000 live births to achieve SDG target of 12 per 1000 live birth, intervention to prevent birth defect and manage them is important [15, 16]. This study can be a basis for National Birth defect Surveillance Registry and thereafter, developing prevention and management guidelines on birth defects. The current study provides evidence on prevalence, patterns, factors and outcomes associated with birth defects across the 12 hospitals in Nepal.

## Method

### Study design and setting

A prospective cohort study was conducted in 12 public hospitals across Nepal to evaluate the efficacy of scale up of Helping Babies Breathe Quality Improvement Project from 1 July 2017 to 17 October 2018 [17]. The total birth in cohort constituted 15% of total birth (home and health facility) taking place in Nepal. The hospitals were selected from different geographic locations across the country, representing different maternal and child health services. Standard ANC services include at least four antenatal check-ups (first at the fourth month, second at the sixth month, third at the eighth month, and fourth at the ninth month of pregnancy); monitoring of blood pressure, weight, and fetal heart rate; provision of information, education, and communication; behavior change communication for danger signs and care during pregnancy; detection and management of complications; and provision of tetanus toxoid immunization, iron tablets, and malaria prophylaxis where necessary. All the hospitals were referral level public hospitals with more than 1000 deliveries per year.

### Study participants

All babies born in the 12 hospitals during the study period were selected for this study. Structural birth defect (cleft lip, cleft lip and palate, congenital heart diseases and defects of gastro intestinal tract or CNS and genetic and chromosomal disorders) categorized as birth defect in the hospital registry was included as birth defect. Stillborn babies, out-born babies and babies whose mothers did not consent or avail themselves were excluded from the study.

### Data collection and management

In the selected hospitals, a data surveillance system, including data collectors and data coordinators, was setup for collection of data on the mothers and newborns. Obstetric data were collected through patient files and maternity register in the maternity wards using a data retrieval form. Socio-demographic data were collected through face-to-face interviews with mothers before discharge. Semi-structured interviews were conducted. Data coordinator assess the completed forms for completeness, which are then indexed, sealed and sent to the head office for further action. In the central office, the data management team, led by a data manager, sort, index, file and reassess for completeness. Data entry operators enter the indexed forms based on the hospitals in Census and Survey Processing System (CSPro). The entered data are cleaned and exported to Statistical Package for the Social Sciences (SPSS) for further data analysis.

### Variables in the study for analysis

Liveborn babies with birth defects were the variable of interest for this study. Demographic variables included age of mother (less than 20 years, 20 to 35 years and 35 years or/and above), ethnicity (brahmin-chettri, relatively advantaged group and others, relatively disadvantaged group), education (literate and illiterate) type of fuel used for cooking in household (clean or polluted) and smoking habit. Antenatal variables included antenatal care (ANC) check-up by doctor/nurse and timing of first ANC visit (first, second or third trimester), parity of mothers (0 previous birth, 1 previous birth and 2 or more previous birth) and severe anemia during pregnancy (7.5 mg/dl). Intrapartum variables included multiple deliveries and sex of the baby. Low birth weight was defined as birth weight less than 2500 g. Birth asphyxia was defined as apgar score less than 7 at 1 min. Neonatal infection was defined as clinical signs of infections at admission to sick newborn care units. Pre-discharge mortality included deaths of newborn with birth defects before discharge.

### Statistical analysis

Prevalence of birth defects was calculated based on the total number of congenital cases reported and total number of live births in the same period. Cross-tabulation was done for socio-demographic, obstetric and neonatal characteristics. Binary logistic regression was performed to analyze the level of association between the characteristics and birth defects. The significance was determined at *p*< 0.05. All variables with *p*< 0.2 in the univariate analysis were considered for multi-variable logistic regression analysis.

### Ethical consideration

All mothers were consented in written before the start of the data collection and confidentiality was maintained. Ethical approval was received from Ethical Review Board of Nepal Health Research Council (reference number 26–2017).

## Results

**Fig. 1:**
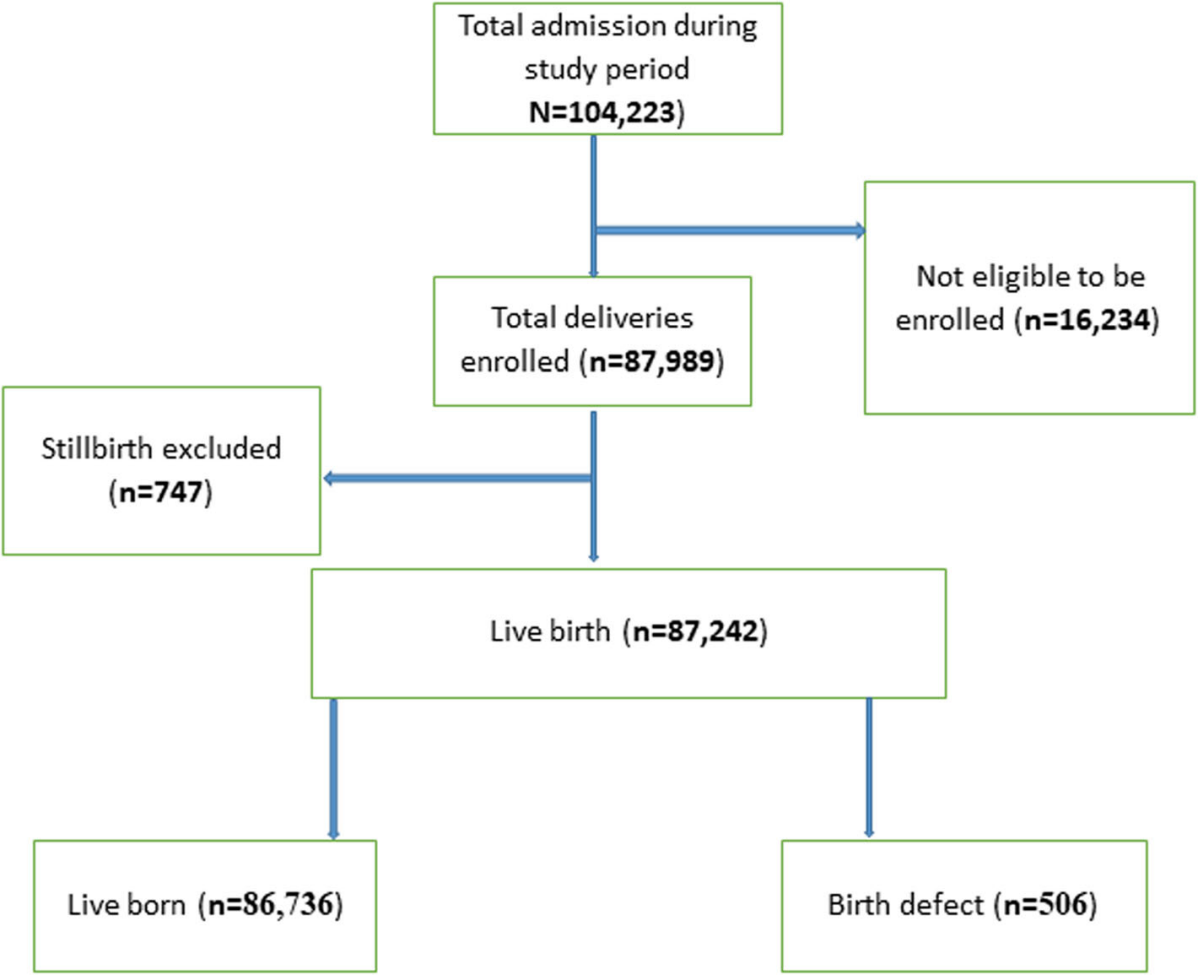
Participants flow figure

**Fig. 2:**
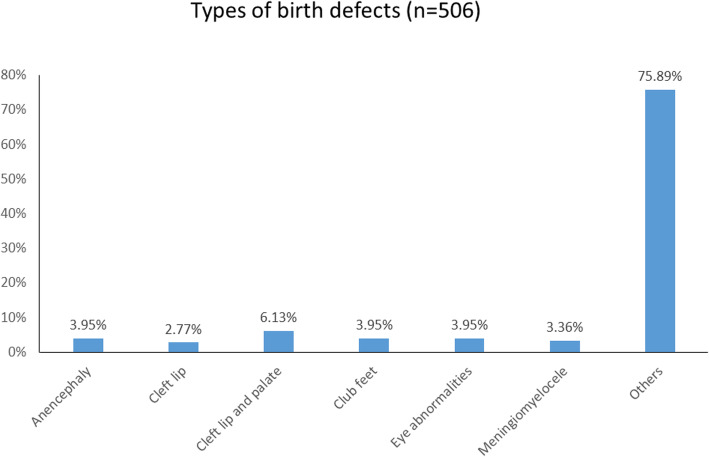
Types of birth defects (*n*=506)

Out of the total 104,223 admissions, a total of 87,989 deliveries were conducted during the study period of which 87,242 deliveries were live births and 747 deliveries were stillbirths. Among them, there were 506 reported cases of birth defects (Fig. 1). The prevalence of birth defects was found to be 5.8 per 1000 live births. The different types of birth defects as reported from the data show anencephaly (3.95%), cleft lip (2.77%), cleft and palate (6.13%), clubfeet (3.95%), eye abnormalities (3.95%) and meningomyelocele (3.36%). Most (75.89%) of the reported cases have not been classified (Fig. 2).

Univariate analysis showed significant association with birth defects for most of the socio-demographic, obstetric and neonatal characteristics. Socio-demographic characteristics such as ethnicity and type of fuel used for cooking in the household showed significant association (< 0.001) with birth defects. Female child was significantly associated with birth defects (*p*=0.002). Women with one previous birth (*p*=0.002) and two or more birth (< 0.001) were significantly associated with birth defects. Multiple delivery was significantly associated with birth defects (*p*=0.02). (Table 1).

**Table 1:** Socio-demographic, obstetric and neonatal characteristics

Variables	Birth defect	No Birth defect	Total	cOR (95% CI)	***p***-value
***Age of mother (n=87,112)***					
< 20 years	47 (9.3%)	6604 (7.6%)	6651 (7.6%)	1.25 (0.93–1.69)	0.15
20–35 years	442 (87.4%)	77,722 (89.7%)	78,164 (89.7%)	Ref	
> 35 years	17 (3.4%)	2280 (2.6%)	2297 (2.6%)	1.31 (0.81–2.13)	0.28
***Literacy (n=66,949)***					
Illiterate	19 (6.0%)	3063 (4.6%)	3082 (4.6%)	1.31 (0.83–2.1)	0.249
Literate	300 (94.0%)	63,567 (95.4%)	63,867 (95.4%)	Ref	
***Ethnicity (n=87,112)***					
Brahmin/Chhetri (relatively advantaged group)	131 (25.9%)	33,897 (39.1%)	34,057 (39.1%)	Ref	
Others (relatively disadvantaged group)	375 (74.1%)	52,709 (60.9%)	53,147 (60.9%)	1.84 (1.51–2.25)	**< 0.001**
***Smoking (n=66,949)***					
No	297 (93.1%)	60,869 (91.4%)	61,166 (91.4%)	Ref	
Yes	22 (6.9%)	5761 (8.6%)	5783 (8.6%)	0.78 (0.51–1.21)	0.268
Type of fuel (*n*=66,722)					
Polluted	142 (45.1%)	16,283 (24.5%)	16,425 (24.6%)	2.53 (2.02–3.16)	**< 0.001**
Clean	173 (54.9%)	50,124 (75.5%)	50,297 (75.4%)	Ref	
***Sex of the baby (n=87,112)***					
Boy	239 (47.2%)	46,836 (54.1%)	47,129 (54.0%)	Ref	
Girl	267 (52.8%)	39,770 (45.9%)	40,037 (46.0%)	1.32 (1.10–1.57)	**0.002**
***Parity (n=87,101)***					
Nullipara	172 (34.1%)	39,882 (46.1%)	40,054 (46.0%)	Ref	
Primipara	183 (36.2%)	30,472 (35.2%)	30,655 (35.2%)	1.39 (1.13–1.72)	**0.002**
Multipara	150 (29.7%)	16,242 (18.8%)	16,392 (18.8%)	2.14 (1.72–2.67)	**< 0.001**
***ANC check-up by doctor/nurse (n=66,949)***					
Yes	316 (99.1%)	66,015 (99.1%)	66,331 (99.1%)	Ref	
No	3 (0.9%)	615 (0.9%)	618 (0.9%)	1.02 (0.33–3.19)	0.974
***Time for first ANC visit (n=66,331)***					
First trimester	189 (59.8%)	29,594 (44.8%)	29,783 (44.9%)	1.68 (1.15–2.46)	0.008
Second trimester	96 (30.4%)	28,276 (42.8%)	28,372 (42.8%)	0.89 (0.59–1.34)	0.58
Third trimester	31 (9.8%)	8145 (12.3%)	8176 (12.3%)	Ref	
***Severe anemia during pregnancy (n=6002)***					
No	28 (96.6%)	5804 (97.2%)	5832 (97.2%)	Ref	
Yes	1 (3.4%)	169 (2.8%)	170 (2.8%)	1.23 (0.17–9.07)	0.841
***Multiple delivery (n=87,112)***					
No	495 (97.8%)	85,667 (98.9%)	86,162 (98.9%)	Ref	
Yes	11 (2.2%)	939 (1.1%)	950 (1.1%)	2.03 (1.11–3.69)	**0.02**

*cOR* crude Odds Ratio

Multivariate analysis showed significant association for various factors with birth defects. Mothers less than 20 years of age were 1.64 times more likely (aOR 1.64; 95% CI, 1.18–2.28, *p*-value=0.003) to be associated with birth defects compared to mothers 20-< 35 years of age. Mothers of ethnicity other than Brahmin/Chhetri were 1.78 times more likely (aOR 1.78; 95% CI, 1.46–2.18, *p* value=< 0.001) to have babies with birth defects. Female child had 1.35-fold risk (aOR 1.35; 95% CI, 1.13–1.61, *p* value < 0.001) of having birth defects compared to male child. Compared to women with no previous birth, the risk of birth defect was higher among women with 1 previous birth (aOR 1.58; 95% CI, 1.26–1.98; *p*< 0.001) and women with 2 or more previous birth (aOR 2.33; 95% CI, 1.84–2.95; *p*< 0.001). Also, mothers with multiple deliveries had 1.8-fold risk of having babies with birth defect compared to mothers with single deliveries (aOR 1.8; 95% CI, 0.98–3.28; *p*=0.06). (Table 2).

**Table 2:** Multivariate analysis of risk factors associated with Birth defects (*n*=66,123)

	aOR (95% C.I.)	p-value
***Age of mother***		
20-< 35 years	Reference	
< 20 years	1.64 (1.18–2.28)	0.003
> 35 years	0.94 (0.57–1.54)	0.79
***Ethnicity***		
Brahmin/Chhetri (relatively advantaged group)	Reference	
Others (relatively disadvantaged group)	1.78 (1.46–2.18)	< 0.001
**Type of fuel**		
Clean		
Polluted	2.26 (1.80–2.84)	< 0.001
***Sex of the baby***		
Boy	Reference	
Girl	1.35 (1.13–1.61)	0.001
***Parity***		
Nullipara	Reference	
Primipara	1.58 (1.26–1.98)	< 0.001
Multipara	2.33 (1.84–2.95)	< 0.001
***Multiple delivery***		
No	Reference	
Yes	1.80 (0.98–3.28)	0.06
***Constant***	0.002	< 0.001

*aOR* adjusted Odds Ratio

In a multi-variable analysis, babies with birth defect have 1.88-fold risk of birth asphyxia (aOR 1.88; 95% CI, 1.41–2.51; *p*< 0.001) in reference with babies without birth defect. Babies with birth defect have 1.82-fold risk of neonatal infection (aOR 1.82; 95% CI, 1.12–2.96; *p*< 0.02). The risk of pre-discharge mortality for babies with birth defects was 3.31 folds (cOR 3.31; 95% CI, 2.13–5.14; p< 0.001) higher compared to babies without any birth defects (Table 3).

**Table 3:** Consequences among babies with birth defects

Variables	Birth defect	No Birth defect	***p***-value	cOR (95% CI)	aOR (95% CI)^**a**^	***p***-value
Low birth weight (*n*=14,676)	99 (0.7%)	Ref	0.18	1.17 (0.93–1.45)	1.15 (0.92–1.44)	0.23
Birth asphyxia (*n*=4971)	53 (1.1%)	Ref	< 0.001	1.94 (1.46–2.59)	1.88 (1.41–2.51)	< 0.001
Neonatal infection (*n*=1462)	17 (1.2%)	Ref	0.004	2.05 (1.26–3.33)	1.82 (1.12–2.96)	0.02
Pre-discharge mortality (*n*=1149)	21 (1.8%)	Ref	< 0.001	3.31 (2.13–5.14)	3.00 (1.93–4.69)	< 0.001

^a^adjusted with age of mother, sex of the baby, parity and multiple delivery

## Discussion

The prevalence of birth defects in the present study is 0.58% which is comparable with the earlier studies in Iraq and Iran which reported an incidence of 0.69 and 0.36% [18–20]. The patterns of system involved in our study show Musculoskeletal System to be most commonly affected presenting as cleft lip, cleft palate and club feet. This was in line with the study conducted in different parts of world in Egypt and India [21, 22]. A study in Iran and India reported higher prevalence of malformations from CNS, Cardiovascular system or Gastrointestinal system [23, 24]. This variation in patterns could possibly be explained by various genetic and environmental factors interplaying differently in varied time and geographical location [10].

Mothers of age group less than 20 years were found to have risk of delivering a newborn with birth defect which was similar to the study which concluded association between young mothers and congenital anomaly [25]. In contrast, several studies have identified the potential of maternal age above 35 years in the causation of this condition [10, 26]. Developmental and behavioural factors like poor diet, illicit drug use, smoking etc. in the adolescent group as compared to older mothers relative to conception could likely affect the developing foetus [25].

According to different literatures, congenital malformation is seen in twins rather than singleton pregnancy [10, 27]. Likewise, our finding enlisted this study as one among those many. However, multiple delivery did not pose a risk to birth defect in Europe [28]. Alterations of the blood flow within the vascular anastomoses supplying the twins and early primary abnormality that might develop during twinning itself can lead to birth defects [29].

In comparison to women with no previous birth, the likelihood of giving birth to a baby with congenital defect was seen among women with 1 or more previous birth in our study, which is similar to those reported in other studies [25, 30]. To the contrary, positive association between nulliparity and range of birth defects have also been successfully investigated earlier [31, 32]. The decrement in body nutrients stores among mothers who have previously delivered as compared to those who have never delivered a baby before explains the association between parity and congenital birth defects [33].

With regards to ethnicity, advantageous ethnic groups like Brahmin and Chettri were comparatively less likely to be associated with birth defects than the non-advantageous group. Association of ethnicity with birth defects has been depicted in the previously done studies [25, 34]. Ethnic variance as the risk for malformations may be linked to genetic susceptiveness or to socio-cultural and economic differences that might modify exposures [35].

This study showed that there was significant impact for female sex in regards to the causation of congenital birth anomalies and this significance is also supported by studies from neighbouring nations [36]. In contrast, male babies were found to have risk for birth defects in another study [36, 37]. This variation could probably be explained by potential of the hormonal hypothesis of sex ratio explaining the unusual concentrations of hormones during or preceding pregnancy could be more common in those malformations with established sex ratio biases [38].

Studies have documented that conditions like cleft lip and polydactyly are more common in males whereas neural tube defects and cleft palate are more seen among female babies [39, 40]. The high incidence of cleft palate and NTDs could also explain female preponderance to for birth defects. However, as we have not examined population-based resources in this study, this might not potentially explain the exact sex difference patterns in babies with congenital birth anomalies.

Babies with birth defects tend to have morbidities such as birth asphyxia and neonatal infection as found in this study. The risk of mortality among these babies at birth is higher similarly. Increased mortality arising from birth defects has also been identified in India [41]. This can be explained based on the type of defect or anomalies which describes the intensity or level of risk associated.

### Limitations

There are some limitations in this study. A community-based study rather than a hospital-based study can better project prevalence and in our settings echocardiography and other advanced diagnostics were not routinely available to diagnose malformations [21]. Further, stillbirths were not included in the study which might also have attributed to the lower prevalence. Birth defects may have been underreported as diagnosis could not have been made at birth. Many birth defects such as congenital heart defects are likely not identified at birth. In a low resource setting of Nepal, it is difficult to classify the exact type of birth defect other than the commonly seen birth defect, so we classified them as “other”.

## Conclusion

Birth defect is prevalent (0.58%) among newborn babies in the same way as reported elsewhere. Various socio-demographic factors like adolescent age and disadvantaged ethnic group are associated with birth defect. Obstetric factors like being female child and number of previous birth are associated with birth defect. Babies with birth defect have high risk for birth asphyxia, neonatal infection and pre-discharge mortality at birth. There is a need to evaluate the services available for babies with birth defect for better identification and management of these babies. Birth defects are major causes of neonatal mortality and morbidity, and national surveillance is very important.

## Data Availability

The datasets used and/or analysed during the current study are available from the corresponding author on reasonable request.

## Abbreviations

LMIC: Low and Middle Income Countries
HIC: High Income Countries
SNCU: Sick newborn care unit
